# Presence and future of plant phenotyping approaches in biostimulant research and development

**DOI:** 10.1093/jxb/erac275

**Published:** 2022-06-30

**Authors:** Nuria De Diego, Lukáš Spíchal

**Affiliations:** Centre of the Region Haná for Biotechnological and Agricultural Research, Czech Advanced Technology and Research Institute (CATRIN), Palacký University Olomouc, Šlechtitelů, Olomouc, Czech Republic; Centre of the Region Haná for Biotechnological and Agricultural Research, Czech Advanced Technology and Research Institute (CATRIN), Palacký University Olomouc, Šlechtitelů, Olomouc, Czech Republic; National Research Council, Italy

**Keywords:** High-throughput screening, mechanism of action, mode of action, -omics, plant biostimulants, plant breeding, plant phenotyping, sensors

## Abstract

Commercial interest in biostimulants as a tool for sustainable green economics and agriculture concepts is on a steep rise, being followed by increasing demand to employ efficient scientific methods to develop new products and understand their mechanisms of action. Biostimulants represent a highly diverse group of agents derived from various natural sources. Regardless of their nutrition content and composition, they are classified by their ability to improve crop performance through enhanced nutrient use efficiency, abiotic stress tolerance, and quality of crops. Numerous reports have described modern, non-invasive sensor-based phenotyping methods in plant research. This review focuses on applying phenotyping approaches in biostimulant research and development, and maps the evolution of interaction of these two intensively growing domains. How phenotyping served to identify new biostimulants, the description of their biological activity, and the mechanism/mode of action are summarized. Special attention is dedicated to the indoor high-throughput methods using model plants suitable for biostimulant screening and developmental pipelines, and high-precision approaches used to determine biostimulant activity. The need for a complex method of testing biostimulants as multicomponent products through integrating other -omic approaches followed by advanced statistical/mathematical tools is emphasized.

## Teaser

High-throughput plant phenotyping can accelerate the development and characterization of new biostimulants. Contrarily, biostimulants can bring new challenges to plant phenotyping to develop complex approaches integrating -omics and data analysis.

## Introduction

‘Plant biostimulants’ is a hypernym used to describe very different substances such as seaweed extracts, humic and fulvic acids, animal- and vegetal-based protein hydrolysates, and microorganisms such as mycorrhizal fungi and rhizospheric bacteria. The definition of plant biostimulants has beeen the subject of extensive discussions and evolution to promote the acceptance of biostimulants by future regulations (du Jardin, 2015; Yakhin *et al.*, 2017; du Jardin *et al.*, 2020). The first legal definition was provided in the US Farm Bill (Agriculture Act of 2018; https://www.congress.gov/115/bills/hr2/BILLS-115hr2enr.pdf) describing a plant biostimulant as ‘a substance or microorganism that, when applied to seeds, plants, or the rhizosphere, stimulates natural processes to enhance or benefit nutrient uptake, nutrient efficiency, tolerance to abiotic stress, or crop quality and yield’. This definition is consistent with the definition of EU Fertilising Products Regulation 2019/1009 describing ‘a plant biostimulant shall be an EU fertilising product the function of which is to stimulate plant nutrition processes independently of the product’s nutrient content with the sole aim of improving one or more of the following characteristics of the plant or the plant rhizosphere: nutrient use efficiency, tolerance to abiotic stress, quality traits, or availability of confined nutrients in the soil or rhizosphere’ (https://eur-lex.europa.eu/legal-content/EN/TXT/PDF/?uri=CELEX:32019R1009&from=EN).

During the last decade, the landscape of the biostimulant industry has changed. Large global companies entered this domain previously occupied mainly by small and medium-sized enterprises. A simple internet web search using the term ‘global biostimulants market size’ offers numerous market reports estimating the average growth at a compound annual growth rate (CAGR) of ~12% during the next 5 years to reach more than US$ 5 billion by 2027. This continually increasing commercial interest in the development and use of biostimulant products, driven by economic and socio-political factors, has been followed by entering the regulatory framework to establish clear standards regarding the claims made by biostimulant producers. For example, in Europe, a CE-certified biostimulant can be placed in the EU Single Market if it fulfills the safety requirements of Regulation (EU) 2019/1009. Besides, the producer must demonstrate plant biostimulant effect to justify the claims following the EU-harmonized standards defined by the Committee for European Standardization (CEN). The biostimulant trials are becoming more demanding and necessitate rigorous and professional practices to fulfill the claims.

Along with biostimulant development, companies have started interacting with the scientific domain and investing significant parts of their revenues into their research and development activities. Scientific approaches that help identify efficient sources for developing new products, clearly and rigorously describe their effects, and, importantly, help understand their mechanism of action are wanted. Testing biostimulant effects in field conditions is highly relevant and unreplaceable; however, some of the claims, such as abiotic stress tolerance, can hardly be justified in the same field trial. In this regard, modern plant phenotyping using non-invasive digital technologies can speed up the development and characterization of the new biostimulants using high-throughput and high-precision approaches in controlled and semi-controlled conditions. They allow multidimensional testing combining different types of application in a broad range of concentrations on plants subjected to several growth conditions, individually or in combination. This review analyzes the evolution of using such approaches in an intensively growing biostimulant research and development domain.

## Plant phenotyping and biostimulants: old terms for modern science

**Fig. 1.**
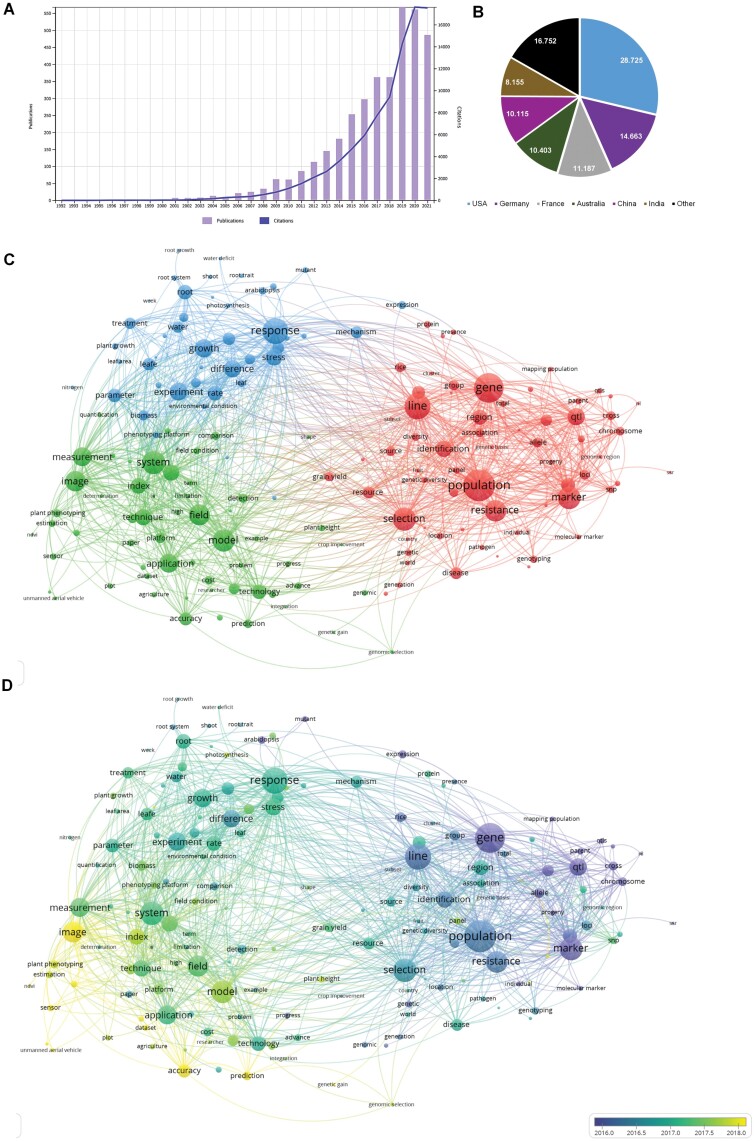
Results related to ‘plant AND phenotyping’ according to the Web of Science using the categories Plant Science, Agronomy, Horticulture, Environmental Science, and Agriculture Multidisciplinary (*n*=3826). (A) Temporal trend in annual numbers of publications (bars) and citations (blue line). (B) Top countries based on authorship affiliations. (C) A network visualization showing three clusters corresponding to different research themes within the field based on the analysis of the co-occurrence of terms in the titles, abstracts, and keywords of the obtained publications. Minimum number of occurrences per node=80. (D) Overlay visualization depicting the evolution of research terms over time.

Literally, ‘plant phenotyping’ refers to a quantitative description of the plant’s anatomical, ontogenetical, physiological, and biochemical properties resulting from genotypic differences and the environmental conditions to which a plant has been exposed. The use of this term in the scientific literature has been evolving. To overview this evolution, we searched under the Basic Search tab on the Web of Science ‘plant AND phenotyping’ in all fields and refined the results by categories; Plant Science, Agronomy, Horticulture, Environmental Science, and Agriculture Multidisciplinary. We obtained 3826 results, including 3255 original articles, 396 review articles, and 171 proceeding papers. The term plant phenotyping was first mentioned in Cenis *et al.* (1992). This study focused on the phenotyping of selected enzymes involved in root-knot nematode symptoms (Fig. 1A). After that, the term ‘phenotyping’ was used more in the sense of its literal definition in plant genetic studies to describe quantitative morphological, biochemical, or biological traits that can be attributed to the marker alleles segregating in a well-defined population as quantitative trait loci (QTL) (Bonierbale *et al.*, 1994; Sripongpangkul *et al.*, 2000). ‘Plant phenotyping’ in the modern sense is a quantitative description of the plant’s anatomical, ontogenetical, physiological, and biochemical traits. The analysis is mainly performed using non-invasive methods to measure plant growth and physiology dynamics over time (Walter *et al.*, 2015). Granier *et al.* (2006) was the first publication using this concept, and the authors presented PHENOPSIS, an automated platform for reproducible phenotyping of plant responses to soil water deficit. Later on, Walter *et al.* (2007) published GROWSCREEN, a novel set up using a simple camera to analyze plant growth. The number of articles presenting plant phenotyping as a non-invasive approach to analyzing plant performance, including morphology and physiology, has reached 579 published articles and 17 593 citations in 2020. To identify research communities historically involved primarily in plant phenotyping, we analyzed the contribution by country/region according to the published affiliations. As shown in Fig. 1B, researchers from the USA were the most prolific publishers, appearing in 28.725% of the publications, followed by Germany and France with 14.663% and 11.187%, respectively.

To go further on the impact of ‘plant phenotyping’ in the research community, we analyzed a corpus consisting of the 3826 obtained publications using VOSviewer V. 1.6.17 (Centre for Science and Technology Studies, University of Leiden) as described by Ruwona and Scherm (2022). This software uses the VOS (visualization of similarities) technique to produce a network map in which the distance among the terms (nodes) reflects their similarities as closely as possible. First, the 3826 publications directly downloaded from the Web of Science were integrated into an Excel file (corpus). The corpus was analyzed for the co-occurrence of terms within titles, abstracts, and keywords, adding the condition of 80 as a minimum number of occurrences per term. The result was a map with three clusters (Fig. 1C); a red cluster contained the highest number of items (66), a blue cluster had 57 items, and a green cluster had 44 items. The red items were dominated by terms related to ‘plant breeding’, containing terms such as ‘population, gene, line, marker, and QTL’, among others. The blue cluster is led by terms such as ‘image, system, technique, technology, model, application, or sensor’, and, to a lesser extent, some terms related to ‘data’ and ‘data analysis’ such as ‘dataset, estimation, and algorithm’. The prevalence terms in the last items (green) were more related to ‘plant response’, including words such as ‘growth, leaf or root’ and ‘environmental conditions’ such as ‘water, drought’, and the model plant ‘*Arabidopsis thaliana*’. Next, the evolution of these terms over time was analyzed using overlay visualization (Fig. 1D). Since the term ‘plant phenotyping’ in publications increased from 2016, most terms appeared in publications in the last 3 years. Terms with early average publication dates (blue and violet, Fig. 1D) predominantly belonged to the first cluster (red in Fig. 1C) focused on plant breeding or scientific studies using Arabidopsis as a plant model. In 2017, the publications related to plant phenotyping changed the focus to experiments using ‘systems’ and ‘techniques’ to measure the plant response to different growth conditions. The latest average publication dates included ‘image’ and ‘sensor’ or terms such as ‘dataset’ and ‘deep learning technologies’ including ‘accuracy and prediction’, pointing to the new ‘sensor to knowledge’ trend in plant phenotyping. Although plant phenotyping is considered an important tool to be used in the plant breeding process (reviewed by Pieruschka and Schurr, 2019), surprisingly, our analyses of the scientific literature show that plant phenotyping is not evolving to support this and that the distance between breeders and scientists is increasing over time (Fig. 1C, D). Several barriers to overcome between plant phenotyping and plant breeding, such as limited seed supply in the segregating generation following the cross, effects of interplant competition, effects of soil and spatial heterogeneity, statistical analysis issues, estimation of genetic gain and response to selection, the relevance of plant (epi)genomics, and automation challenges, were in this context named by Fasoula *et al.* (2020). Recently published papers reporting the use of phenotyping in genome-wide association studies (GWAS) and transcriptome-wide association studies (TWAS) are good indicators that this trend will not be long lasting (Yang *et al.*, 2014; Chawade *et al.*, 2019; Li *et al.*, 2020; Ferguson *et al.*, 2021).

**Fig. 2.**
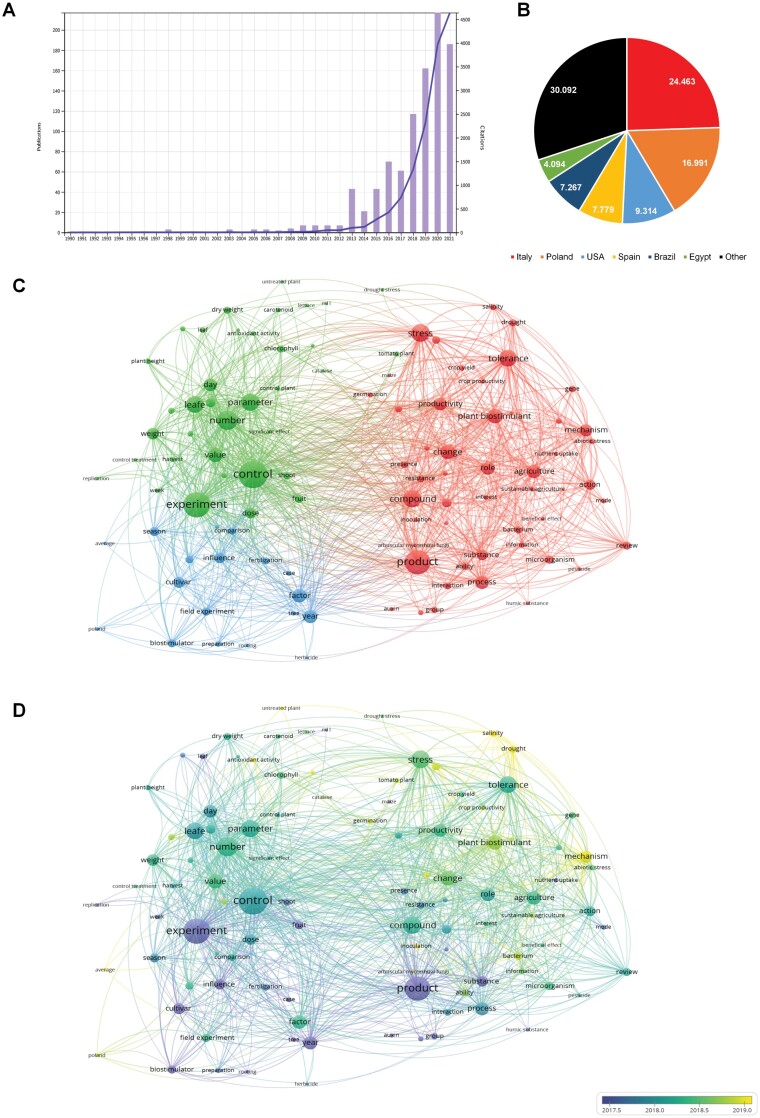
Results related to ‘plant AND biostimulant OR biostimulator’ according to the Web of Science using the categories Plant Science, Agronomy, Horticulture, Environmental Science, and Agriculture Multidisciplinary (*n*=977). (A) Temporal trend in annual numbers of publications (bars) and citations (blue line). (B) Top countries based on authorship affiliations. (C) A network visualization showing three clusters corresponding to different research themes within the field based on the analysis of the co-occurrence of terms in the titles, abstracts, and keywords of the obtained publications. Minimum number of occurrences per node=30. (D) Overlay visualization depicting the evolution of research terms over time.

We searched under the Basic Search tab of the Web of Science ‘plant AND biostimulant OR biostimulator’ using the same criteria. We obtained 977 results, including 750 articles, 98 review articles, and 111 proceedings papers. The first use of the term ‘plant biostimulant’ appeared in Sanders *et al.* (1990) (Fig. 2A). This study focused on the effect of two commercial biostimulants (Enersol from humic acids and Ergostim from folic acid) in carrot emergence and root development. As the main results, the authors showed that the exogenous application of both biostimulants improved seedling emergence and root weight. At this time, the term ‘biostimulant’ or ‘biostimulator’ was used to define various substances, including synthetic molecules such as the phytohormone 6-benzylaminopurine or the fungicide propiconazole (Goatley and Schmidt, 1991). As observed in Fig. 2A, the scientific interest in biostimulants started a bit later than in the case of plant phenotyping, and the number of publications per year (~5) did not increase until 2008. In this year, the publications and citations related to plant biostimulants increased exponentially. In 2020 the terms ‘plant AND biostimulant OR biostimulator’ were recorded in 217 articles and 3969 citations. Of all these publications, 24.463% came from scientists with Italian affiliation, followed by 16.991% and 9.314% from Poland and the USA, respectively (Fig. 2B). It is worth mentioning that over the years, the definition of biostimulant evolved, and recently in both North America and Europe, the definition has been unified and is legally binding (as described in the Introduction).

The 977 publications related to plant biostimulants were also analyzed using VOSviewer. Due to the lower number of publications, the minimum number of occurrences per term was reduced to 30. Again a map formed by three main clusters was obtained: a red cluster contained almost half of the items (49), a blue cluster with 36 items, and a green cluster with 18 items (Fig. 2C). The red items were dominated by ‘product, compound, or plant biostimulant’ and other terms such as ‘stress, tolerance, mechanism, and agriculture’. The second item (green) was formed by terms such as ‘control and experiment’ and many others related to morphological and physiological traits such as ‘parameters, weight, value, dry weight, chlorophyll, or antioxidant capacity’. The last items (blue) contained unrelated terms such as factor, year, cultivar, influence, season, etc. When the overlay visualization was performed, we observed that the average publication dates for these terms appear from 2017 to 2019. The first mentioned terms are mainly ‘product, experiment’ or, in lower numbers, ‘substance, biostimulator, influence, or fertilization’ (violet). Most of the terms have an average publication date in 2018 (blue to green). They include terms related to the biostimulant itself, such as ‘compound, role, action’, and the experimental set up such as ‘control, number, parameters, stress, tolerance’, among others, showing that this year was the most relevant for the evolution of the keywords in plant biostimulant research. The latest terms were ‘mechanism, drought, drought stress, salt, and untreated plants’. Similar to the previous analysis, we could observe a fast evolution in plant biostimulant research, starting with introducing new products and substances mainly for fertilization of the plants through a more exhaustive study of the plant response at morphological and physiological levels to end in the description of the mechanism. Many of these studies are reporting successful use of a biostimulant application in the early stages of a plant’s development (Amooaghaie and Moghym, 2011; Khan *et al.*, 2017; Batool *et al.*, 2020). However, recent studies were focused on the biostimulant effect on plant production under different conditions (Khan *et al.*, 2021; Rashid et al., 2021a, b; Jiménez-Arias *et al.*, 2022). Interestingly, in the VOSviewer analysis, no term strictly related to plant phenotyping appears in any of the three items on the map.

## Plant phenotyping and biostimulants: trendy scientific topics without a connection

**Fig. 3.**
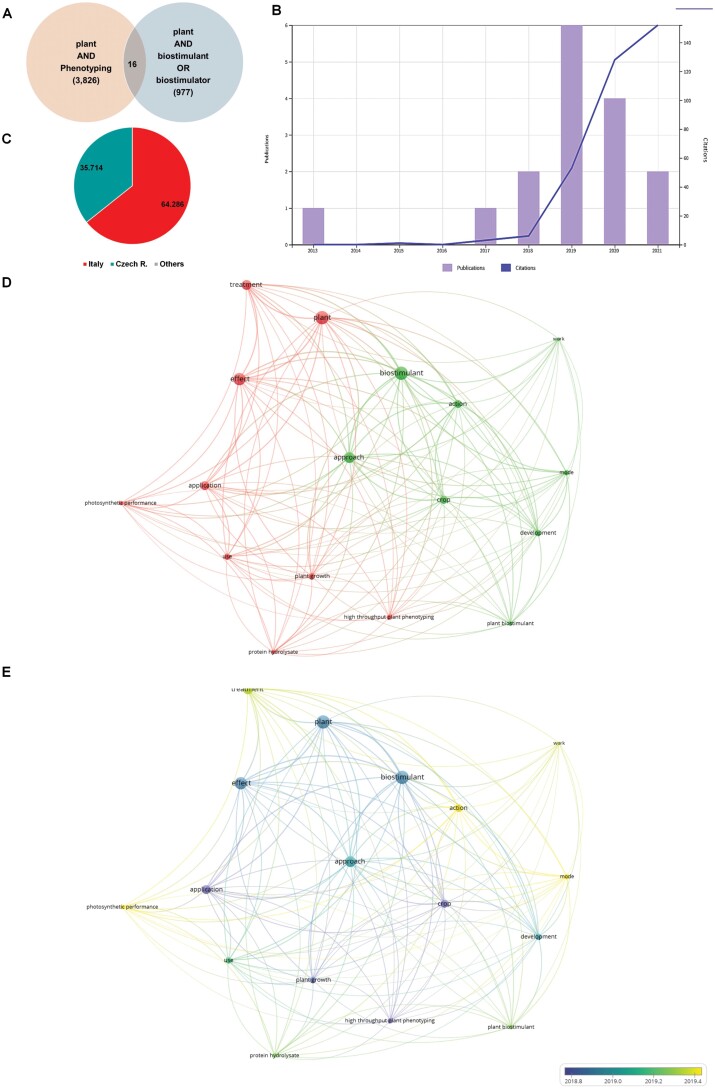
Results related to ‘plant AND phenotyping AND biostimulant’ according to the Web of Science using the categories Plant Science, Agronomy, Horticulture, Environmental Science, and Agriculture Multidisciplinary (*n*=16). (A) Venn diagram representing the publications related to plant phenotyping and/or plant biostimulants. (B) Temporal trend in annual numbers of publications (bars) and citations (blue line). (C) Top countries based on authorship affiliations. (D) A network visualization showing three clusters corresponding to different research themes within the field based on the analysis of the co-occurrence of terms in the titles, abstracts, and keywords of the obtained publications. Minimum number of occurrences per node=4. (E) Overlay visualization depicting the evolution of research terms over time.

Plant phenotyping and biostimulants generated interest in the scientific communities simultaneously. However, no interconnections between both terms appear in the VOS analysis. To evaluate the degree of connection between both scientific communities, we performed a new search on the Web of Science using ‘plant AND phenotyping AND biostimulant OR biostimulator’ as keywords. We found only 16 results, comprising 12 original works and four review articles (Fig. 3A, B; Tables 1, 2). More than half of the publications originate from scientists with Italian affiliation, followed by the Czech Republic with 31.25% (Fig. 3C). The first publication integrating both scientific topics appeared in 2012 as an opinion article published in *Acta Horticulturae* by Summerer *et al.* (2013) resulting from the I. World Congress on the Use of Biostimulants in Agriculture organized in Strasbourg (France) (Table 1). There, the authors pointed to high-throughput phenotyping as a more efficient approach than traditional bioassays for simultaneous comparison of the inducing potential of chemical substances (i.e. hormones) with molecules of proven biostimulant activity. They also described three sensors as non-invasive methods for uncovering the biostimulants’ effects on plants. During this congress, an additional two studies as proceeding papers were presented in which the terms ‘phenomics AND biostimulants’ (but not ‘phenotyping’) appeared together (Petrozza et al., 2012a, b). The two studies are an excellent example of studying the biostimulant effect on plants (in this case, tomatoes) using the described sensors. However, the first original research integrating the topics ‘plant AND phenotyping AND biostimulant OR biostimulator’ as keywords appeared 3 years later, with the highest number of publications (six) and citations (123) recorded in 2019 and 2020, respectively (Fig. 3B).

**Table 1.** Review articles related to ‘plant AND phenotyping AND biostimulant’ according to the Web of Science using the categories Plant Science, Agronomy, Horticulture, Environmental Science, and Agriculture Multidisciplinary

Title	Journal	Reference
High-throughput plant phenotyping: a new and objective method to detect and analyze the biostimulant properties of different products	*Acta Horticulturae*	Summerer *et al.* (2013)
Applications of seaweed extracts in Australian agriculture: past, present and future	*Journal of Applied Phycology*	Arioli *et al.* (2015)
High-throughput plant phenotyping for developing novel biostimulants: from lab to field or from field to lab?	*Frontiers in Plant Science*	Rouphael *et al.* (2018)
Algae biostimulants: a critical look at microalgal biostimulants for sustainable agricultural practices	*Biotechnology Advances*	Kapoore *et al.* (2021)

**Table 2.** Original articles related to ‘plant AND phenotyping AND biostimulant’ according to the Web of Science using the categories Plant Science, Agronomy, Horticulture, Environmental Science, and Agriculture Multidisciplinary

Plant species	Biostimulants	Application	Growth conditions	Sensors	Interesting traits	Other -omics	References
*Arabidopsis thaliana* L. and *Eragrostis tef*	800 natural products and their derivatives	Culture medium	Control conditions	Side- and top-view RGB (microphenotron)	Root and shoot growth	–	Burrell *et al.* (2017)
*Arabidopsis thaliana* L. and six varieties of *Brassica napus* L.	*Ascophyllum nodosum*	Spraying	Control conditions	RGB	Reduce pod shatter and yield loss	–	Łangowski et al. (2019)
*Arabidopsis thaliana* L.	Eleven protein hydrolysates	Seed priming	Control conditions or salinity	RGB (PlantScreen™ XYZ System, PSI) and FluorCam (PlantScreen™ Compact System, PSI)	Plant growth, fluorescence-related parameters, and PBC index	Untargeted metabolomics	Sorrentino *et al.* (2021a)
*Capsicum annuum* L.	Seaweed extract (CL-SW) or metabolite formula (ICL-NewFo1)	Irrigation	Control conditions or drought	Lysimeters Plantarray 3.0 platform (Plant-Ditech)	Control conditions: CL-SW increased transpiration, biomass, and yield Drought: ICL-NewFo1 delayed water loss	–	Dalal *et al.* 2019
*Solanum lycopersicum* L.	Five prototypes (Valagro)	Drenching	Drought	RGB (Scanalyzer 3D system; LemnaTec GmbH, Aachen, Germany)	All improved digital biomass and water-use efficiency		Danzi *et al.* (2019)
*Solanum lycopersicum* L.	Eight protein hydrolysates	Spraying	Control conditions	RGB (top and side view) and FluorCam (PlantScreen™ Modular System, PSI)	Two products improved root growth rate and growth performance	Untargeted metabolomics	Paul *et al.* (2019a)
*Solanum lycopersicum* L.	One protein hydrolysate	Spraying or drenching	Control conditions or drought	RGB (top and side-view) and FluorCam (PlantScreen™ Modular System, PSI)	Drenching better than foliar application. Increased digital biomass and transpiration	Untargeted metabolomics	Paul *et al.* (2019b)
*Solanum lycopersicum* L.	Commercial glycine betaine	Spraying	Control conditions or Drought	Semi-automated multi-chamber whole-canopy system	Photosynthesis, transpiration, and water-use efficiency	Untargeted metabolomics	Antonucci *et al.* (2021)
*Solanum lycopersicum* L.	18 Crude bio-extracts (CBEs) obtained from microalgae and cyanobacteria	Drenching	Control conditions	Root and shoot length using a ruler	Root and shoot biomass, N, P, and K uptake	Targeted metabolomics	Mutale-joan *et al.* (2020)
*Triticum turgidum* L. subsp. *durum* (Desf) Husn.	Thyme essential oil or *Paraburkholderia phytofirmans* (PsJN)	Seed coating	Control conditions	MultiSpeQ PhotosynQ platform (http://www.photosynq.org)	Control conditions: PsJN increased biomass, leaf thickness, and photosynthesis. Water–nutrient stress: different strategies; thyme essential oil maintained water balance and PsJN leaf thickness and photosynthesis		Ben-Jabeur *et al.* (2021)
*Zea mays* L. cv. Ronaldinho	Plant growth-promoting rhizobacterium [*Bacillus licheniformis* (FMCH001)]	Seed coating	Control conditions or drought	Soil water content and crop coverage. Conveyor system (ProInvent A/S, Hørsholm, Denmark)	Root and shoot dry weight, WUE, and catalase activity		Akhtar et al . (2020)
*Zea mays* L., hybrid P0423, Pioneer or *Glycine max* L. Merr.	Three prototypes	Spraying	Control conditions	RGB and FluorCam (Scanalyzer 3D system, LemnaTec GmbH)	All improved digital biomass and green area	Genomics	Briglia *et al.* (2019)
–	Lactic acid bacteria and rhizobacteria	–	Growth medium	Near-infrared (NIR) and UV-visible-NIR (UV-Vis-NIR) spectroscopy	Selection of the interesting bacteria		Treguier *et al.* (2021)

The 16 identified publications using ‘plant AND phenotyping AND biostimulant OR biostimulator’ as keywords were analyzed using VOSviewer to understand the actual state of the art of plant phenotyping and biostimulant connection. Due to the low number of works, this time, the minimum number of occurrences was reduced to four, ending up with 17 items divided into two clusters; red (nine) and green (eight) (Fig. 3D). The first item (red) is formed by terms such as ‘plant, treatment, application, use, high throughput phenotyping’ and parameters such as ‘plant growth and chlorophyll fluorescence’ and ‘protein hydrolysate’ as the only type of plant biostimulants. The second item (green) is mainly led by ‘biostimulant’ followed by ‘approach, action, crop, development, mode, work, and plant biostimulant’. In addition, the overlay visualization showed that almost all of these terms appeared in the publications at the same time (2018) (Fig. 3E), whereas the average publication date for the terms ‘treatment, mode, and action’ is 2019. These results showed that the studies focused on plant biostimulants using phenotyping approaches are still in development and far from being connected to the plant phenotyping research communities. However, we believe that these technologies can speed up the selection of new products and help understand their mechanism of action described in the perspective article published by Rouphael *et al.* (2018) (Table 1).

Another critical point nowadays is the evolution of the biostimulant-based products, so they are more focused on complex substances (i.e. seaweed extracts, humic and fulvic acids, animal- and plant-based protein hydrolysates, or formulations that includes microorganisms such as mycorrhizal fungi and rhizospheric bacteria) than on simple natural molecules (i.e. plant hormones or specific amino acids). Thus, the complexity of the new biostimulants due to their natural origin (i.e. seaweeds), the raw material (i.e. animal- and plant-based protein hydrolysates), and/or the preparation procedure needs an in-depth study to understand not only their mode of action but also the stability of the batches and viability of the final products. Plant phenotyping has been identified as a beneficial technology for simultaneously testing different batches, extraction processes, and final products, thanks to the high-throughput screening (HTS) approaches (Rouphael *et al.*, 2018).

## Indoor phenotyping for plant biostimulant screening and study of the mechanism of action

This section presents insight into the possible use of phenotyping approaches for HTS in biostimulant development. Further, we provide an overview of how indoor phenotyping systems equipped with different sensors were used to describe the effect of plant biostimulant application on traits of interest, pointing to a potential mechanism of action (Table 2).

### High-throughput phenotype-based screening approaches using Arabidopsis as a model plant

The first original work was published in 2017 by Burrell *et al.* The authors introduced a HTS approach for testing libraries of chemical or natural compounds based on *in vitro* Arabidopsis growth, in which the root and shoot biomass is quantified using simple pictures performed by red–blue–green (RGB) cameras installed in a microphenotron (cabinet) (Burrell *et al.*, 2017). Arabidopsis is a good plant model for screening processes because of its small size and short life cycle. In a microphenotron, a group of Arabidopsis seeds is sown into phytostrips with eight wells each. Then, 12 phytostrips are installed in a 96-well microtiter plate. The cabinet allows the simultaneous growth of 27 microtitre plates, giving a total of 2592 seedlings. Due to the small dimensions of the plates, growth can be recorded only for a maximum of 11 d. One limitation of this approach is the difficulty in homogenizing the number of seeds per well due to the tiny size of the Arabidopsis seeds. This fact reduces the accuracy and increases the experimental error of the method. One solution could be to use an appropriate software routine for counting the seed number to normalize the results, as shown by Ugena *et al.* (2018). Another option is using HTS approaches based on the rosette growth of very young Arabidopsis seedlings individually placed in 48-well plates (Ugena *et al.*, 2018; Sorrentino *et al.*, 2021a; Hernándiz *et al.*, 2022). This method is performed in a growth chamber in which an XYZ robotic arm is automatically moved above the plates to take RGB images of single plates (more details in De Diego *et al.*, 2017). The advantage of this methodology is that it can simultaneously analyze 572 plates (counting 27 456 seedlings). This means that this ultra-HTS approach allows the simultaneous testing of many compounds from different natural sources at a wide range of concentrations in Arabidopsis seedlings grown under different growth conditions, including osmotic stress, salt stress, or nutritional stress (De Diego *et al.*, 2017; Bryksová *et al.*, 2020).

Additionally, simple RGB imaging offers the extraction of many traits (e.g. rosette growth and plant greenness) that can be integrated to calculate a unique number called the Plant Biostimulant Characterization (PBC) index for a more straightforward classification of biostimulants as plant growth promotors and/or stress alleviators, or growth inhibitors (Ugena *et al.*, 2018; Sorrentino *et al.*, 2021a). Additionally, Sorrentino *et al.* (2021a) demonstrate the high reproducibility of this ultra-HTS approach for comparing biostimulants from different raw materials. The characterization of plant biostimulants using ultra-HTS approaches also gives a high number of biological replicates (seedlings per variant), which provides enough material to be used in the complementary analysis (i.e. metabolomics) (Sorrentino *et al.*, 2021a). Additionally, the analysis based on Arabidopsis growth has been successful not only in characterizing single compounds or substances but also in studying the impact of the beneficial microorganisms on plant performance and stress response (Sánchez-López *et al.*, 2016; González-Pérez *et al.*, 2018; Fan *et al.*, 2020). However, the number of works describing the use of HTS methods employing model plants such as Arabidopsis for biostimulant screening are today still minimal. HTS technologies are accessible at those phenotyping installations with controlled conditions, mainly localized at scientific institutions, and represent only a minor part of the existing systems. Their accessibility to the biostimulant industry is thus limited.

Moreover, even if they are equipped with simple RGB sensors, professional automated phenotyping platforms can be too expensive for smaller companies. As a solution, several studies have presented low-cost systems for plant phenotyping (Bagley *et al.*, 2020; Ribes *et al.*, 2020; Wu *et al.*, 2020). However, such systems can serve well for scientific purposes but do not necessarily need to meet the routine and standardized screening campaigns called for in industrial practices. From a future perspective, developing more affordable systems and/or devices allowing high-throughput phenotype-based screening with a simple readout using model plants, suitable to be integrated into the biostimulant industries’ pipelines, are needed.

### Phenotyping approaches to study the mechanism of action of plant biostimulants

Yakhin *et al.* (2017) clearly described the need to distinguish between determining the ‘mode of action’ and ‘mechanism of action’ in order to understand the function of a complex multicomponent product such as a biostimulant. They suggested that ‘the focus of biostimulant research and validation should be upon proof of efficacy and safety and the determination of a broad mechanism of action, without a requirement for a specific mode of action’ (Yakhin *et al.*, 2017). For that, high-precision phenotyping methods employing multiple sensors can be beneficial using model plants or directly using crops of interest. In general, indoor plant phenotyping presents the advantage of control conditions that can be repeated over experiments (Rouphael *et al.*, 2018). Moreover, the biostimulant effects that can hardly be studied in the field conditions, such as abiotic stress tolerance, can be easily performed there. As shown in Table 2, among the crops of the highest agronomical interest, tomato (*Solanum lycopersicum* L.) is the favorite species for studying biostimulants using plant phenotyping methods. In tomato plants, biostimulants from different sources [complex biostimulants based on a crude bioextract obtained from microalgae or cyanobacteria (Mutale-joan *et al.*, 2020), eight protein hydrolysates from different plant species (Paul *et al.*, 2019a), a commercial product (Petrozza *et al.*, 2014), or a simple product based on glycine betaine (Antonucci *et al.*, 2021)] improved plant growth (Table 2). In another work, the foliar spray of three prototypes prepared from seaweed or plant extracts with selected micronutrients improved the digital mass and the green area of *Zea mays* L., hybrid P0423, Pioneer, and *Glycine max* L. Merr. (Briglia *et al.*, 2019).

On the other hand, the application of seaweed-based biostimulant (CL-SW) or a prepared formulation (ICL-NewFo1) in peppers (*Capsicum annuum* L.) showed contrasting results: CL-SW improved plant transpiration, biomass, and final yield under control conditions, whereas ICL-NewFo1 delayed water loss under drought stress (Dalal *et al.*, 2019). Coated seeds of *Triticum turgidum* L. subsp. *durum* (Desf) Husn. with thyme essential oil or the endophyte *Paraburkholderia phytofirmans* (PsJN) activated different strategies in the plants to deal with the water–nutrient stress (Ben-Jabeur *et al.*, 2021). While the plants primed with thyme essential oil maintained water balance, those treated with PsJN increased their leaf thickness and photosynthesis. The type of application in which a biostimulant is tested can also influence plant performance (Table 2). Drenching with a particular substance based on protein hydrolysates enhanced tomato growth (measured as digital biomass) and transpiration under control and stress conditions compared with foliar application (Paul *et al.*, 2019b). The biostimulant sources and type of application thus condition the response of the plants, which at the same time depends on the species and growth conditions.

Contrasting methods of analysis, ranging from manual measurement using a ruler to fully automated approaches using multiple sensors, can be found in the literature describing the analysis of the effect of biostimulant application on plants (Paul *et al.*, 2019a; Mutale-joan *et al.*, 2020) (Table 2). However, nowadays, the most preferred plant phenotyping methods are the non-destructive ones that permit the kinetic analysis of the plant’s performance (Humplík *et al.*, 2015); they are much faster and offer simultaneous analysis of a much higher number of individuals. Phenotyping methods based on RBG imaging are the most used to characterize the effects of plant biostimulants, appearing in seven of the 12 original articles reported in Table 2. These have been successfully used to evaluate the biostimulant effect in plants from very early developmental stages to production (Ugena *et al.*, 2018; Łangowski *et al.*, 2019). However, for a further understanding of the physiological effects, the combination of RGB with FluorCam for measuring the fluorescence-related parameters (light phase of plant photosynthesis) permits a deeper analysis of the response of the plants treated with plant biostimulants (Briglia *et al.*, 2019; Sorrentino *et al.*, 2021a). Using lysimeters can be very helpful for water deficit studies, directly informing on the plants’ water loss (Dalal *et al.*, 2019) (Table 2). Alternatively, infrared gas exchange equipment connected to a multichamber whole-canopy system was presented to be used to measure the biostimulant effect on the photosynthetic-related parameters (dark phase) and the plant water use efficiency in tomatoes (Antonucci *et al.*, 2021). More sophisticated sensors (hyperspectral cameras) have also been used for characterizing biostimulants based on beneficial microorganisms. For example, these were used to identify the most efficient *Trichoderma* spp. strains able to constrain *Rhizoctonia solani* Kuhn, *Sclerotinia sclerotiorum* (Lib.) de Bary, and *Sclerotium rolfsii* Sacc. growth *in vitro* and to study the effect of these microbes on plants *in vivo* using baby lettuce as a host (Manganiello *et al.*, 2021). Near-infrared (NIR) and UV-visible-NIR (UV-Vis-NIR) spectroscopy was also used for selecting lactic acid bacteria and rhizobacteria *in vitro* (Treguier *et al.*, 2021).

## Understanding of biostimulant mechanism/mode of action through the integration of other -omics and advanced statistical tools

Well-determined selection of sensor(s) or their combination for the phenotyping experiment can offer the appropriate tool for characterizing the biostimulant’s mechanism(s) of action. Even if biostimulants are mostly complex multicomponent products, we believe that advanced plant phenotyping combined with various -omics approaches followed by data analysis using advanced statistical tools can be used to unravel the potential biostimulant mode of action. Untargeted metabolomics was shown to be a valuable technique to be combined with phenomics for characterizing the potential mode of action of different biostimulants applied to Arabidopsis (Sorrentino *et al.*, 2021a), tomato (Paul et al., 2019a, b; Antonucci *et al.*, 2021; Sorrentino *et al.*, 2021b) or lettuce (Sorrentino *et al.*, 2021b). The challenging part of combining both -omics is the accumulation of a considerable amount of data, requiring suitable tools for analyzing the big datasets. The use of multivariate statistical approaches (frequently employed in metabolomics) allows the classification of the scores (variants) and uncovers the main metabolic differences between untreated and treated plants. Sorrentino *et al.* (2021b) compared the effect of the same biostimulants in two crop species (tomato and lettuce) and discovered different species-specific responses in control and stress conditions. In this case, the machine learning algorithm defined as random forest classification pointed in tomato to traits related to chlorophyll fluorescence parameters in combination with specific antistress metabolites that benefit the electron transport chain, such as 4-hydroxycoumarin and vitamin K1 (phylloquinone). In lettuce, biomass-related parameters and water use efficiency were the most relevant traits, with a better response connected mainly to plant hormone regulation, especially auxins (Sorrentino *et al.*, 2021b).

Similarly, the effect of three biostimulants in *Z. mays* L., hybrid P0423, Pioneer, and *G. max* L. Merr. was studied using a combination of phenotyping with next-generation sequencing (NGS), revealing activation of different species-specific responses (Briglia *et al.*, 2019). In maize, genes involved in hormone (cytokinin and auxin) metabolism/catabolism, maltose biosynthesis, sugar transport, and phloem loading were up-regulated. In contrast, genes involved in nitrogen metabolism, metal ion transport (mainly zinc and iron), sulfate reduction, and amino acid biosynthesis were up-regulated in soybean. Mathematical tools can also represent essential improvements in the HTS campaigns to identify new biostimulants. A good example is the work by Treguier *et al.* (2021), where artificial neural network models were presented as tools to correctly identify the genus (species) of 70% (63%) of the lactic acid bacteria and 67% of the rhizobacteria on an independent prediction set of unknown bacterial strains. The integration of plant phenotyping with other -omics can provide additional value to the experimental set ups offering the next step for a deeper understanding of the mode of action of plant biostimulants. Advanced mathematical tools can further contribute to identifying new traits, markers, or metabolic pathways beneficial for the discovery, evaluation, and development of new biostimulants.

## Conclusions

Plant phenotyping and biostimulants represent dynamically evolving domains in research and development of the last decade. Interestingly, they show the opposite supply–demand interaction of science and industry. Plant phenotyping technologies appeared on demand from scientists to satisfy their needs for more efficient ways to describe plant phenotypes. On the other hand, industrial producers of biostimulants recognized a need to interact with the scientific domain and searched for new technologies to improve their research and development. Our analysis of the scientific literature showed the evolution of the trends in both fields with recognizable recent and future ‘meeting points’. (i) Plant phenotyping: plant breeding (QTL) → new non-invasive systems to phenotype plants data analysis and deep learning. (ii) Biostimulants: testing products in simple assays → new parameters studied under controlled conditions or stress → mechanism of action in plants under salt and drought conditions. (iii) Plant phenotyping and biostimulants: new approaches for testing crop development induced by biostimulants → type of treatment and their mode of action.

**Fig. 4.**
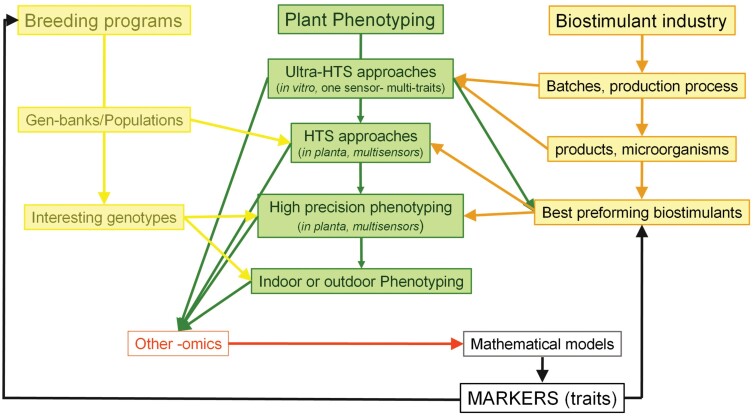
Scheme representing the suggested interconnection between ‘plant breeding’, ‘plant phenotyping’, and ‘biostimulants’.

Practically, plant phenotyping technologies seem to become essential for the biostimulant industry. They can significantly contribute to: (i) basic research of biostimulants, and characterization of their mechanism/mode of action; (ii) screening campaigns for the identification of new biostimulants from new raw materials and beneficial microorganisms; (iii) developmental pipelines for analyses of batches and testing of a new method of preparation; (iv) production pipelines for shelf-life analysis and quality control; and (v) justification of the claims requested by certification authorities,

On the contrary, testing biostimulants as complex multicomponent products can stimulate the need to develop new complex phenotyping approaches and methodologies integrating different -omics and robust data analysis tools such as multivariate statistical approaches and artificial intelligence. Multidimensional biostimulant testing combining different application types (seed, foliar, and soil) in broad concentration ranges in multiple environmental conditions requires a high-throughput capacity for testing hardware and analysis software. Higher testing capacities of phenotyping facilities and/or development of affordable devices will be needed for high-throughput phenotype-based screening and data analysis ready for integration into the biostimulant R&D industries’ pipelines. Moreover, we believe that plant phenotyping should reconnect to plant breeding. By doing so, it will open a new perspective of biostimulant testing, offering the possibility of developing targeted biostimulants with a specific activity in crops grown in conditions for which they were primarily selected by breeding programs. An ideal interconnection among the plant phenotyping scientific community, biostimulant industry, and plant breeders is presented in Fig. 4. Ultra-HTS is routinely used to test new technologies related to the final products’ production, batches, stability, and shelf-life. Additionally, the new products are tested in model plants under different growth conditions and, at the same time, compared with well-established products with confirmed activity for faster selection. Once selected, the new products are tested in different crops/varieties/genotypes, preferably suggested by breeders (depending on the interest) under different growth conditions using HTS approaches (early developmental stages). For further information, the studies can be combined with other -omics (e.g. genomics and metabolomics) to better understand the mechanism of action. Finally, the results are evaluated using an integrative plant phenotyping approach indoors and/or outdoors in the plant species of interest. All data must be analyzed using advanced statistical/mathematical tools (machine learning/artificial intelligence) for identifying relevant markers (phenotyping traits) to simplify the pipeline for the selection of new interesting ‘genotypes’ and ‘biostimulants’ together. Hence, genotypes showing a positive response to certain biostimulants can also be selected.

## Data Availability

The complete list of the source publications used for VOSviewer analyses presented in this publication are available from the corresponding author, Lukáš Spíchal, upon request.
